# The (Mis)use of Psychotropic Drugs and Addiction to Anxiolytics among Older Adults Living at Home or in Retirement Homes: Implications for Quality of Life

**DOI:** 10.3390/healthcare11212908

**Published:** 2023-11-06

**Authors:** Mirjana Kralj, Krešimir Šolić, Robert Lovrić

**Affiliations:** 1 Faculty of Medicine, Josip Juraj Strossmayer University of Osijek, 31000 Osijek, Croatia; mkvasilj@fdmz.hr (M.K.); kresimir@mefos.hr (K.Š.); 2 Nursing Institute “Professor Radivoje Radić”, Faculty of Dental Medicine and Health Osijek, Josip Juraj Strossmayer University of Osijek, 31000 Osijek, Croatia

**Keywords:** older adults, multimorbidity, polypharmacy, quality of life, psychotropic drugs

## Abstract

Nowadays, the growing number of people aged 65+ has become a global phenomenon. At that age, the most common medical problems are multimorbidity and inappropriate polypharmacy, which have a negative impact on the quality of life in older adults. The aim of this cross-sectional study was to examine comorbidity, the use of psychopharmaceuticals, and symptoms of addiction to anxiolytics among older adults living at home or in retirement homes, and to examine the differences in quality of life in relation to the use and misuse of psychotropic drugs. The research included 383 people aged 65+ living in the Republic of Croatia (EU). A standardized questionnaire CAGE was used to collect data about the use of psychotropic drugs. Quality of life was examined using the WHOQOL-BREF scale. The average age of respondents was 83 years. There is a significantly higher prevalence of anxiety disorders (*p* = 0.001) in respondents who live at home. Psychopharmaceuticals were used by 218 (56.9%) respondents, equally in both groups of respondents. A total of 77 (20.1%) respondents had been using anxiolytics for more than five years, while 26 (6.8%) of them had significant clinical symptoms of addiction to anxiolytics. All domains and the overall quality of life scale were significantly lower (*p* < 0.001) in respondents who have clinical symptoms of anxiolytic addiction. The results indicate that the use of psychotropic drugs by respondents is inappropriate. Respondents who inappropriately and excessively use psychotropic drugs have a significantly worse quality of life.

## 1. Introduction

Life expectancy is on the rise, leading to a growing proportion of older adults (aged 65 and older) [1]. According to the Croatian Bureau of Statistics, the percentage of older adults in Croatia is 19.2% [2]. By the year 2050, the percentage of older adults is estimated to grow to 26.8% [3]; one out of every three citizens will be aged 65+, and one out of every ten citizens will be aged 80+ [3].

This growing population of older adults is likely to experience multimorbidity, which negatively impacts their physical and mental health and consequently decrease their quality of life [4,5] as their abilities no longer meet the demands of modern life [6,7]. Krizmanić and Kolesarić [8] define quality of life as the subjective perception of one’s own life determined by the objective circumstances a person lives in and their personality, which have an impact on reality perception and specific life experience.

Multimorbidity, the simultaneous presence of several chronic conditions in older adults, leads to the prolonged usage of various medications, approximately four to five of which are prescription and at least two of which are used in self-medication [9]. As a result, polypharmacy, the simultaneous usage of many medications, occurs regularly in older adults, even though it is often unnecessary. Additionally, the pharmacokinetic and pharmacodynamic characteristics of medications can be different in older adults [10,11]. Polypharmacy compounds these challenges and significantly increases the risk of adverse side effects and the interactions of medications. The frequency of hospitalization due to pharmacotherapy significantly decreases the quality of life of older adults and is accompanied by unnecessary healthcare costs [10].

A major issue for older adults taking medication is the lack of proper adherence, failure to take medications according to the instructions, and self-medication [12]. Improper medication use in older adults is often characterized by unintentional or intentional consumption of smaller or larger doses of medication than prescribed, especially psychopharmaceuticals [13,14]. This nonadherence can be caused by various factors such as age, financial constraints, misunderstanding medication instructions, methods, and regimen, as well as functional and sensory limitations in older adults [15]. Another significant factor contributing to nonadherence and poor adherence is the absence of perceived benefits, i.e., visible treatment outcomes. Ultimately, excessive use of psychopharmaceuticals among older adults increases healthcare costs [10].

An additional challenge for older adults is the prevalence of anxiety disorders among this population [16]. In older adults, anxiety can develop as a reaction to a stressful event (recent mourning), as a symptom of a bad adaptation to an environment or life conditions (sense of loneliness, nonconformity, hearing disorder) or it can mean the development of a serious emotional disease (personal or family history of depression) [16,17]. Moreover, it can develop as a side effect of a somatic disease (chronic pain, inability, dementia) or as a side effect of prescribed medications. As older adults are reluctant to admit to having psychological problems and their families and physicians may misidentify the aforementioned problems due to common prejudices about aging processes or life stresses and losses, it is unfortunately very common that anxiety often remains unrecognized and untreated.

The symptoms of untreated anxiety disorder can worsen over time, eventually leading to depression in older adults. This can manifest in very serious symptoms, including a depressed mood, feelings of guilt, and the emergence of thoughts related to self-harm or suicide [16,17]. Depression and anxiety in older adults lead to the loss of or a reduction in functional abilities, which affects life satisfaction and results in lower quality of life [18,19].

Psychopharmaceuticals, including antidepressants, antipsychotics, mood stabilizers, anxiolytics, and hypnotics, are employed in the pharmacotherapeutic treatment of anxiety and depression in older adults. Adherence of psychotropic drugs among older adults is essential. This involves initiating treatment with the lowest anticipated therapeutic dose (considerably lower than therapeutic doses for middle-aged and younger individuals), and then gradually increasing the chosen drug’s dosage while monitoring clinical improvement, aiming to reach the lowest effective dose [14,15,16,17]. It is important to steer clear of medications with potential sedative effects and to avoid combinations of drugs that could result in complications. Additionally, it is essential to consistently and frequently monitor for potential side effects [14,15,16,17].

For a longer period, there has been a growing trend in the consumption of various psychotropic medications by older adults without any confirmation of a mental disorder [20]. Numerous studies show high risks of their usage above the permitted dose, as well as other irregularities in prescribing them [21,22,23,24]. The most commonly used and misused medications are painkillers, antibiotics, and anxiolytics [16]. One alarming trend is the frequency of inappropriate and excessive prescription of benzodiazepine to older adults.

Increased consumption of psychotropic medications could lead to complications, long-term consumption, comorbidity with other diseases, physiological changes at old age, unwanted reactions, and addiction [25,26]. Addiction is usually defined as forced activity and irresistible engagement of some specific action [27], usually in the form of consumption of certain substances, where this consumption is neither medically nor nutritionally justified. Despite a slight decline in the use of anxiolytics among older adults in European countries in recent years (except in Slovakia, Spain, and Portugal) [28], anxiolytics remain among the most commonly prescribed and abused medications [29,30]. In Croatia, the prescribing of anxiolytics has been spiraling out of control, often involving inappropriately high dosages or prolonged use [28]. In fact, in 2021, a staggering 4,718,583 prescriptions for anxiolytics were issued, constituting 8.5% of all prescribed [31]. In older adults, the dependency syndrome can manifest through psychomotor disturbances, loss of self-control, impaired speech, pronounced drowsiness, cognitive difficulties, and more [32]. Continued use of anxiolytics in older adults can lead to intoxication, and in severe cases, even death [32]. The use of low doses of anxiolytics in older adults can also result in addiction, which significantly depends on various factors such as health status, physiological, pathological, and pharmacological changes associated with aging [32]. Abrupt cessation of anxiolytic use in older adults can trigger withdrawal symptoms, known as withdrawal syndrome. Due to the increased consumption of psychotropic medications, many countries prescribe guidelines for the psychopharmaceutical treatment of older adults [20,28,29]. 

It is obvious that the use and misuse of psychotropic medications, their side effects, addiction, and their impact on the quality of life of older adults have become a global problem. 

Therefore, the aim of this study was to examine the prevalence of diseases (comorbidity), the use of psychotropic drugs, and symptoms of addiction to anxiolytics between older adults living at home and those living in retirement home, and to examine the differences in their quality of life in relation to their (mis)use of psychotropic medications.

## 2. Materials and Methods

### 2.1. Study Design

A six-month cross-sectional study was conducted in four primary care physician’s offices and four retirement homes in Osijek-Baranja County, Republic of Croatia. 

### 2.2. Respondents

The study included older adults aged 65+ living in Osijek-Baranja County. The first group included respondents living in their own home, whereas the second group included respondents living in retirement homes. Respondents were recruited using the “face-to-face” method, which proved to be a very efficient recruitment strategy in older adults [33]. Respondents were recruited by researchers (authors of this article) in the mentioned research institutions whose employees provided support and cooperation. 

Immediately before data collection, the researchers (authors of this article) explained to respondents all the details of the study (aim, methods, ethical issues, anonymity, expected contribution of the study, etc.), upon which the respondents signed the informed consent to voluntarily participate in the study. The recruitment process was based on inclusion criteria which were defined in accordance with the study’s aims and design. The inclusion criteria were as follows: age 65+, voluntary participation, understanding Croatian language, ability to communicate verbally, health condition that enables undisturbed participation in this study. The inclusion criteria for the institutions were (a) geographic region of Osijek-Baranja County, Republic of Croatia; (b) health institution belonging to the category of primary healthcare according to the Croatian classification; (c) retirement home specialized in care for older adults; (d) approval of competent institutional committee for conducting the study. The recruitment process continued during the study through the procedures of explaining all details of the study to potential respondents, obtaining their informed consent to participate in the study, adhering to all ethical standards, and supporting the respondents during the study, which is further described in the article. 

The sample size was calculated using G*Power software (version 3.1.2, F. Faul, University of Kiel, Kiel, Germany). To observe a medium effect in the difference of numerical variables, with a significance level of 0.05 and a power of 0.8, the minimum required sample size was found to be 128 respondents, i.e., 64 per group. 

### 2.3. Instruments

The data were collected using a three-part questionnaire. The first part of the questionnaire contained 11 items on general information and health status of the respondents (age, gender, place of residence, marital status, level of education, demographic and socio-economic status). Also, the first part of the questionnaire contained six items about health status and usage of psychotropic medications. 

The second part of the questionnaire was a structured instrument CAGE (C—Cutting Down; A—Annoyance by Criticism; G—Guilty Feeling; E—Eye-openers) for the self-evaluation of symptoms of addiction [34], which is free to use for researchers. The questionnaire, an acronym of Cut, Annoyed, Guilty, Eye-opener, was originally used for the self-evaluation of symptoms of addiction to alcohol [34]. CAGE contains four questions in total, which were previously translated into the Croatian language and are often used in healthcare institutions in Croatia for the assessment of various addictions (alcohol, medications, drugs) [35]. This four-item instrument examines the quantity of taken medications (multiple choice, yes/no answers) with a 5-point range scale. 

The sum of points higher than two indicates more frequent usage and clinically significant symptoms of addiction to these medications. In order to meet the need of this study the questionnaire was modified to examine respondents’ self-evaluation of the symptoms of addiction to anxiolytics [34].

The third part of the questionnaire was a structured instrument of the World Health Organization for the evaluation of quality of life, Quality of Life (WHOQOL-BREF), which is also free to use for researchers. Psychometric analysis showed WHOQOL-BREF to be a reliable and valid instrument [36,37]. All items and domains of WHOQOL scale were previously translated into the Croatian language and psychometrically validated in the Croatian context [38]. The instrument estimates the subjective perception of an individual’s quality of life within 4 weeks in the context of their culture, system of values, personal goals, standards, and care. The instrument contains 26 items, which examine four life domains (physical health, mental health, social relationships, and environment). The result of each domain is an average of the described items assessed using a 5-point Likert scale (1= least agreement with the statement, 5 = highest agreement with the statement). The range of the total score Is from 26 to 100, where a higher score indicates better quality of life. 

Prior to the main study, a pilot study was conducted on 24 randomly selected respondents who did not participate in the main study (12 older adults from the examined health institutions and 12 older adults from the examined retirement homes) in order to further check the clarity and comprehensibility of the questionnaire [39]. 

### 2.4. Data Collection

In the first respondents’ group, data were collected during their visits to their primary care physician’s office in Osijek-Baranja county, as well as from the data given by the respondents themselves. Consent to conduct the study was obtained from the supervisors of four general primary care physician’s offices and four retirement homes. Before completing the questionnaire, the researchers provided additional explanations about the content and how to complete it to the respondents. The researchers were always available to clarify any possible ambiguity offering advice and additional explanations (clarifying the questions, way of marking the answers, interpreting the meaning of the Likert scale answers, etc.) without any personal suggestions while providing answers. Prior to completing the structured questionnaire, the specific data were collected by analyzing respondents’ medical records: exact name of medications, length of usage, prescription, usage of psychotropic medications (anxiolytics, sedatives, hypnotics), data about physical and mental diseases, complaints, symptoms, etc. Furthermore, data were collected by using the described instruments to estimate the attitude towards medications and the quality of life.

Data from the second group of respondents, who lived in retirement homes, were collected using the previously described instruments. It was possible for the respondents to complete the questionnaires in the institutions’ dining halls at a time of their choosing. Each respondent was given a pen and paper, the content of the questionnaire was explained, and any necessary help was provided. Data were also collected from their medical records available at their primary care physician’s office. Completion of the questionnaire for both respondents’ groups was anonymous and not time-bound.

### 2.5. Data Analysis

Categorical data were presented as absolute and relative frequencies. Numerical data were described by median and the limits of the interquartile range. Differences in categorical variables were tested using χ^2^ test and, if necessary, Fisher’s exact test. The normality of distribution of the numerical data was tested using the Shapiro–Wilk test. The differences of numerical variables between the two independent groups were tested using the Mann–Whitney U test. Multivariate regression analysis was used to identify predictors of worse perceived quality of life. All P values are double-sided. The level of significance is set at Alpha = 0.05. The statistical program MedCalc^®^ Statistical Software version 20.123 (MedCalc Software Ltd., Ostend, Belgium) was used for the statistical analysis.

### 2.6. Ethical Considerations

Prior to data collection, the researchers thoroughly informed the respondents about the objectives and other details of the study, ethical questions, and details of the questionnaire. Respondents had the right to withdraw from the study at any time. The permission of the ethical committee was obtained from the competent higher education institution (IRB approval number: 2158-61-07-15-12). Collected data were used for the purpose of this study and did not violate respondents’ privacy or data confidentiality in any way.

## 3. Results

### 3.1. Sociodemographic Characteristics of Respondents

The study included 383 respondents, 86 (22.5%) male and 297 (77.5%) female respondents. The median age of respondents was 79 years (IQR = 72–84) (Table 1). The median age of female respondents (80 years; IQR = 73–85) was significantly higher (*p* = 0.033) than the median age of male respondents (74 years; IQR = 68–83). There were 189 (49.3%) respondents living in retirement homes, and 194 (50.7%) respondents living in their own home. Most of them, 214 (55.9%), were widowed. Most of the respondents, 145 (37.9%), have finished secondary education, while 118 (30.8%) respondents had finished elementary education 118 (Table 1).

### 3.2. Disease Prevalence among Respondents (Comorbidity)

Rheumatic diseases were most common, present in 245 (64%) respondents, followed by cardiovascular diseases, present in 234 (61.1%) respondents. Insomnia was the most common mental illness. A total of 114 (29.8%) respondents had anxiety disorders, which were more common in respondents living at home in relation to the respondents living in a retirement home (*p* = 0.001) (Table 2).

### 3.3. Frequency of Use of Psychotropic Drugs

A total of 218 (56.9%) respondents used psychotropic drugs, and 117 (30.5%) respondents occasionally used anxiolytics. The medications were usually prescribed by primary care physicians. A total of 77 (20.1%) respondents used anxiolytics and there was no significant difference in place of residence. A total of 154 (40.2%) respondents had regular medical examinations. (Table 3).

### 3.4. Prevalence of Clinically Significant Symptoms of Addiction to Anxiolytics (CAGE Scale) 

Significantly more respondents living in a retirement home felt the need to reduce the amount of tranquilizers and sleep medications they use (*p* = 0.01) than those living at home. Also, they were offended when other people warned them about potential issues with taking medications (*p* = 0.008). As many as 354 (92.4%) respondents expressed feeling bad or guilty for using tranquilizers. A total of 271 respondents (70.8%) admitted to sometimes taking tranquilizers first thing upon waking up (Table 4).

Respondents who had clinically significant symptoms of addiction to anxiolytics used psychotropic drugs significantly more often and had consumed them for longer than five years (*p* < 0.001). Furthermore, respondents who had clinically significant symptoms of addiction underwent medical examination occasionally or regularly (*p* = 0.02) (Table 5).

### 3.5. Perceived Quality of Life of Respondents (WHOQOL Scale)

According to the WHOQOL scale, the median perceived quality of life in all respondents was 59 (IQR 47–69), (range 26–100). According to the use of anxiolytics, there was a significant difference between female and male respondents living in retirement homes (Table 6). Female respondents showed significantly higher perception of their physical health (*p* = 0.02), mental health (*p* = 0.01), social functioning (*p* = 0.004), and overall quality of life (*p* = 0.003) than male respondents. The results showed no significant differences in relation to gender according to use of anxiolytics in respondents living at home (Table 6).

Respondents who had clinically significant symptoms of addiction to anxiolytics perceived their quality of life to be much worse in relation to respondents with no clinical symptoms in all domains of WHOQOL scale: physical and mental health (*p* < 0.001), social functioning (*p* = 0.003), environment (*p* < 0.001), overall scale of quality of life (*p* < 0.001) (Table 7).

Respondents who live at home and have clinically significant symptoms of addiction to anxiolytics perceived their quality of life to be much worse in relation to the respondents with no clinical symptoms in all domains of WHOQOL scale, except environment: physical (*p* = 0.007) and mental health (*p* < 0.002), social functioning (*p* = 0.003), overall scale of quality of life (*p* < 0.006). Similarly, respondents living in a retirement home and having clinically significant symptoms of addiction to anxiolytics perceived their quality of life to be much worse than respondents with no clinical symptoms in all domains of WHOQOL scale, apart from social functioning and environment: physical (*p* = 0.004) and mental health (*p* < 0.001), overall scale of quality of life (*p* < 0.02). (Table 8).

Multivariate logistic regression was used to examine the predictors of worse perceived quality of life in respondents. The median was 58.6, and lower scores were considered a poorer quality of life (Table 9).

Symptoms of addiction (CAGE) (OR = 2.63), 3–6 months long use of anxiolytics (OR = 2.30), and medications prescribed by psychiatrist (OR = 3.66) were the predictors of increased likelihood of poorer quality of life in all respondents. Secondary level of education (OR = 0.59) and no use of psychopharmaceuticals (OR = 0.56) were the predictors of decreased likelihood of worse perceived quality of life.

Significant symptoms of addiction (CAGE) (OR = 3.1), 3–6 months long use of anxiolytics (OR = 17.5) and medications prescribed by psychiatrist (OR = 4.87) were the predictors of increased likelihood of worse perceived quality of life in the group of respondents living at home. Secondary level of education was a predictor of decreased likelihood of worse perceived quality of life (OR = 0.27).

Significant symptoms of addiction (CAGE) (OR = 2.98), 3–6 months long use of anxiolytics (OR = 11.9) or use of anxiolytics for more than 5 years (OR = 2.54), and medications prescribed by psychiatrist (OR = 4.54) were the predictors of increased likelihood of worse perceived quality of life in respondents living in retirement home (Table 9).

## 4. Discussion

The aim of this study was to examine the prevalence of the most common mental illnesses and the frequency of consumption of psychotropic drugs between the two groups of respondents, living at home and living in a retirement home. Furthermore, the study examined the differences in prevalence of clinically significant symptoms of addiction to anxiolytics between the two mentioned groups of respondents. Finally, the study examined the perceived quality of life of respondents and the differences in the quality of life in relation to the (mis)use of psychotropic drugs.

### 4.1. Sociodemographic Characteristics of Respondents

This study included significantly more female than male respondents, which was anticipated due to higher life expectancy for women (79.9 years) than for men (73.4 years) in the Republic of Croatia and worldwide [40]. The results showed that more than half of the respondents are widowed or without a partner. This is supported by other studies on the impact of changes in family and society that affect older adults’ social environment [3]. The last few decades have brought huge and sudden changes in the lifestyle and makeup of families, as well as an increase in the number of older adults put in an institution. The formerly traditional, patriarchal, and mostly rural family was replaced by a new, industrially developed, and shaped family [41]. These changes have led to new, numerous problems which consequently had a great impact on older adults. In the past, large multigenerational families provided their older members with appropriate support and complete care. Nowadays, modern families face numerous difficulties and problems when providing care and support for older members. At the same time, there are fewer and fewer members in modern families who can provide quality care for older members. Thus, the complete care and support of older family members is being transferred to various forms of care and support outside the family in institutions specializing in the care of older adults [41]. The results of this study showed that only 13.2% of the respondents living in a retirement home have a spouse. There has been a global increase in the number of residential facilities for older adults [41]. There is a growing need for this type of permanent housing due to the inability of older adults to look after themselves or to be looked after by other, usually employed, family members. That is why a permanent residential facility is often the only and most dominant form of care for older adults [42].

### 4.2. Diseases Prevalence among Respondents (Comorbidity)

It is well known that older adults suffer from multimorbidity, i.e., simultaneous presence of several chronic conditions in an individual, which leads to usage of higher number of medications, approximately four to five of which are prescription and at least two of which are self-medication [23]. Whatever form of multimorbidity of chronic diseases there are, from the patient’s point of view, it means simultaneous treatment of these diseases and use of several types of medications. Therefore, multimorbidity is the main cause and risk of polypharmacy [23].

When it comes to comorbid physical illnesses, this study’s results showed that 64% of respondents suffered from rheumatic diseases, while 61.1% suffered from heart and blood vessel diseases. The most common mental diseases were insomnia, in 37.6% of respondents; and anxiety disorders, in 29.8% of respondents. Insomnia is defined as having some troubles with falling asleep, keeping the continuity of sleep, or waking up too soon [42]. The most prominent risk factors for the onset of insomnia are age and gender. The reason for higher prevalence of insomnia in older adults is not exactly determined [42,43]. It is believed that aging results in a partial decline in the functionality of the sleep control system. One of the most certain factors which increases the frequency of insomnia is the presence of numerous comorbidity conditions. As a result of insomnia, older adults have numerous difficulties in everyday functioning, which have a negative effect on their quality of life. It must be acknowledged that insomnia could be a symptom of other diseases or mental disorders. The harmful consequences of long-term insomnia are reflected in difficult, sometimes impossible daily functioning, and mood swings. Due to sleepiness and reduced attention, the risk of falls and injuries increases, which further causes disruption in family relationships [42,44].

The fact that multimorbidity is related to the patient’s age was confirmed by many epidemiological studies, which state that old age brings additional risks for the development of more chronic diseases [45]. The connection between gender and the prevalence of multimorbidity was also proven, showing that women of older age and poorer socioeconomic status have a significantly higher risk of developing multimorbidity. The risk is even higher in mental disease patients [46].

### 4.3. Frequency of Use of Psychotropic Drugs and Anxiolytics, Drug Prescription, and Frequency of Medical Examination

This study’s results showed that psychotropic drugs were used by almost 57% of the respondents, and anxiolytics by more than 30%. The high percentage of use of psychotropic drugs is caused by inappropriate and excessive prescription. Other studies found that 70% of older patients were treated well with these groups of medications [21,47]. Psychopharmacological treatment of older adults requires careful determination of symptoms and knowledge of the health status of each elderly patient, as well as adherence to new treatment guidelines [48,49].

A study conducted in the public university hospital in Norway reported that a long-term consumption of addictive medications, which are mostly prescribed for pain and insomnia (especially benzodiazepines and opioids), significantly complicates the treatment of disease, and leads to extensive complications in patients aged 65 to 90 [45]. Other studies conducted in the USA warn that benzodiazepines are the most common group of medications prescribed to older adults over 65 years of age, primarily for treatment of anxiety and insomnia [19,43].

A big study conducted in West Virginia, which included 98,970 respondents aged 65+, showed that women are significantly more likely to be prescribed benzodiazepines than men, and almost three times as many are prescribed to patients with one form of dementia [50]. There are numerous other problems that lead to improper medication intake, such as mistakes in the regularity of medication intake by older adults, and unintentional or intentional intake of smaller or larger doses than prescribed [13].

According to the results of this study, respondents living in a retirement home felt significantly more often the need to reduce their use of sedatives and/or sleep medications. Also, they felt significantly more offended if people pointed out their medication intake issues. These results are in accordance with the Belgian study conducted among retirement home residents, which shows that benzodiazepines were used by 52.4% of respondents inappropriately and excessively in a period longer than a year to treat the symptoms of insomnia and anxiety [51].

According to the definition by the World Health Organization, rational and recommended pharmacology implies therapeutically correct and economically profitable use of medications by professionals and consumers. The prevalent inappropriate use of medications adversely impacts overall healthcare costs, challenges the quality of medication supply, contributes to antimicrobial resistance, and raises concerns about treatment outcomes [47]. Other negative effects are manifested in an increased risk of adverse reactions to drugs, mutual interactions of taken drugs and many other side effects, especially expressed in older adults [52]. Due to all these reasons, caring for patients suffering from several chronic diseases is extremely demanding. This study showed that medications were mostly prescribed by primary care physicians, in as many as 86.6% cases, which implies a large availability of medications, an increase in consumption, and a higher risk of misuse. Primary care physicians, as the first point of contact in the healthcare system, play a crucial role in prescribing psychotropic drugs for older adults. This role has certain advantages, such as quick and easy accessibility of medications, the possibility of continuous therapy adjustment according to the needs of older adults, monitoring medication effectiveness, reducing the workload of specialized services of psychiatrists, etc. However, it is also important for primary care physicians to be well-educated in order to avoid potential risks, including errors in symptom recognition and treatment of mental disorders, prescribing medications without appropriate indications, inadequate patient medication adherence monitoring, potential drug interactions and misuse, insufficient management of adverse effects or patient deterioration, and the issue of stigmatization, among others. Numerous studies indicate high risks of consumption above permitted doses and other irregularities dealing with prescribing medications [22,24].

Given the above, it is important to study, evaluate, and apply prescribing strategies, (i.e., rational prescribing and deprescribing of psychotropic drugs), and avoiding prolonged use whenever it is clinically unnecessary [53]. Due to the ever-increasing use of psychopharmacological drugs, which has numerous and serious consequences, many countries prescribe guidelines for the psychopharmacological treatment of older adults [17,26].

### 4.4. Prevalence of Clinical Symptoms of Addiction to Anxiolytics (CAGE Scale)

The results of this study showed that respondents with significant symptoms of addiction to anxiolytics used psychotropic drugs more often, used anxiolytics perpetually, and consumed them consistently for longer than five years. A study conducted in Pennsylvania had similar results: 80% of respondents had problems with inappropriate and simultaneous usage of benzodiazepines and opioids, risk of developing addiction, and higher risk of overdose [53]. Other studies report excessive and irrational consumption of these medications and insufficient control by physicians [54,55]. This is supported by the results of this study, which clearly showed that 24% of respondents did not go to medical examinations. Various characteristics of aging, such as the weakening of physical and mental abilities, loss of autonomy, and reduced ability to satisfy biological and social needs, increase the risk of developing various diseases, including the risk of mental disorders. Most often, these are anxiety disorders. Although there are not many differences in anxiety between older and younger people [56,57], in old age, anxiety has its own symptoms, such as the fear of falling and resulting trauma [58]. The consequences of untreated anxiety in older adults can lead to cognitive disorders, lower quality of life, problems with their environment, alcohol addiction, and many other types of impairment to their physical, mental, and social health [27].

### 4.5. Perceived Quality of Life of Respondents (WHOQOL Scale)

According to the WHOQOL scale, the median perceived quality of life of all respondents was 59, (range 26–100).

The experience of life in such facilities for older is extremely subjective. Despite the objective factors of the retirement home environment in which an individual lives, the individual perception of these same factors determines the subjective quality of life. Some studies describe that the mental and physical health of older adults are largely related to their own self-concept, which leads to a higher level of satisfaction with the quality of their own life [59,60,61]. Satisfaction with the quality of life is defined as cognitive evaluation of the subjective perception of one’s welfare. It differs from the emotional component, even though they are tightly connected [14]. The results of other studies describe the increase in life satisfaction among people aged between 40 and 70 years, whereas it declines in the later period of life [62].

According to the use of anxiolytics, there was a significant difference between female and male respondents living in retirement homes. Female respondents showed significantly higher perception of their physical health, mental health, social functioning, and overall quality of life than male respondents. The results of this study showed a significant difference in perceived quality of life between male and female respondents living at home, whereas female respondents living in a retirement home perceived their quality of life to be much higher than male respondents in all domains, as well as in the total WHOQOL scale. These results can be interpreted on the assumption that women often feel useful by providing help to other home residents and they are much more active in physical and social activities where they outnumber male residents. Presumably, such behavior can result in a better mood and sense of one’s own contribution to other people’s lives. This is supported by the results of the study conducted in the Republic of Croatia among older women who, regardless of being single, still have close people they can rely on and maintain social contacts. Also, they do not feel rejected by the community or family [63].

Respondents having clinically significant symptoms of addiction to anxiolytics perceived their quality of life to be much worse than the respondents with no clinical symptoms. This result was anticipated due to addiction symptoms which relate to various issues (physical symptoms and problems, weaker cognitive functions, emotional difficulties followed by the feelings of loneliness, inferiority, etc.). Therefore, it is obvious that various symptoms of addiction to anxiolytics endanger the everyday quality of life of the respondents. Unfortunately, due to aforementioned facts, older adults get caught in a vicious cycle of additional diagnostic and therapeutic procedures, and increasing pharmacotherapy, which adversely affects their quality of life.

Previously mentioned results, supported by the results of multivariate regression analysis, imply that the predictors of worse perceived quality of life are significant symptoms of addiction to anxiolytics, their use for more than 3 months, and the use of medications prescribed by psychiatrists in both respondents’ groups. These results should be taken seriously and considered when planning strategies and methods to prevent uncontrolled and inappropriate use of anxiolytics in older adults, as well as when planning strategies and methods to improve their quality of life.

### 4.6. Limitations of the Study

There are several limitations of this study. The first one refers to the possibility of selecting respondents, i.e., the lack of possibility to talk to a person with impaired communicative and cognitive abilities, or a person with some serious mental disorders and other pathological conditions. Also, this was a cross-sectional study, and the determination of direct cause and effect relationships between variables could not be established. Moreover, although the method of data collection was an anonymous questionnaire, there was a possibility that the respondents answered some questions by giving some socially acceptable answers.

### 4.7. Usefulness and Applicability of Study Results

This study reflects the uniqueness of the obtained data for the researched field. Also, it will be an incentive for healthcare professionals and scientists to conduct new international comparative studies. Furthermore, the results of this study are a clear indicator of the need to design convincing and efficient methods to reduce the consumption of psychotropic drugs in older adults, and thus to reduce the negative consequences of their inappropriate use. Moreover, it is necessary to advance interventions and control mechanisms in all phases of the process, from prescribing psychotropic drugs (who, when, why, how much, and in what way), to acquisition, and finally to the use of prescribed medication, which is very important. It is crucial to activate various services in all areas of care for older adults, especially in primary healthcare and home care, as well as older adults’ families, neighbors, and friends. Additionally, modern technology should be leveraged further to design new or adapt existing computer programs and available mobile applications which will then be used by physicians, pharmacists, and even older adults for planning, calculating, keeping records, and controlling prescription, acquisition, and use of psychotropic drugs (method, time, quantity, dosage, etc.). Mechanisms to control and record medication effects and possible unwanted reactions and side effects should also be improved. Such holistic and individualized approaches to control and record procedures for all stages could significantly contribute to the efficient prescription and use of psychotropic drugs in older adults. In the long term, the applicability of these results can be manifested in the development of new strategies to improve the quality of life in older adults through social and family support, which will ultimately lead to a reduction in overall treatment costs.

## 5. Conclusions

The results of this study lead to the conclusion that anxiolytics, sedatives, and hypnotics are usually prescribed by primary care physicians. The respondents from both groups, living at home or in a retirement home, use psychotropic drugs excessively and without any control due to insufficient cooperation with physicians.

Respondents in both groups who inadequately and excessively use psychotropic drugs and have significant symptoms of addiction to anxiolytics perceive their quality of life to be significantly worse than those without clinical symptoms. The use of anxiolytics for more than 3 months and medications prescribed by psychiatrists were also identified as predictors of worse perceived quality of life in both groups of respondents.

There is an evident need to introduce well-organized and effective regulations on the use of psychotropic drugs in older adults to improve their quality of life. This century is the century of older adults due to the rapid and unrelenting increase in the share of this population in society. As a result, people of the third age are becoming the dominant group in the population structure, and as such, should be given particular attention.

## Figures and Tables

**Table 1:** Sociodemographic characteristics of respondents (n = 383).

Characteristics	Place of Residence		
	Home n = 194	Retirement Home n = 189	Total n = 383
	n (%)	n (%)	n (%)
Gender			
male	62 (32)	24 (12.7)	86 (22.5)
female	132 (68)	165 (87.3)	297 (77.5)
Place of residence			
city	101 (52.1)	0	101 (26.4)
suburb	22 (11.3)	0	22 (5.7)
village	71 (37.2)	0	71 (37.2)
institution	0	189 (100)	189 (100)
Family status			
married	94 (48.5)	25 (13.2)	119 (31.1)
widowed	85 (43.8)	129 (68.3)	214 (55.9)
divorced	9 (4.6)	13 (6.9)	22 (5.7)
single	6 (3.1)	22 (11.6)	28 (7.3)
Level of education			
none	33 (17)	46 (24.3)	79 (20.6)
primary	65 (33.5)	53 (28)	118 (30.8)
secondary	77 (39.7)	68 (36)	145 (37.9)
tertiary	19 (9.8)	22 (11.6)	41 (51.2)
Age/years: median (IQR)	74 (69–81)	83 (77–87)	79 (72–84)
Total	194 (100)	189 (100)	383 (100)

**Table 2:** Prevalence of accompanying diseases in respondents living at home vs. in retirement homes.

Accompanying Diseases	Place of Residence			*p* Value *
	Home n = 194	Retirement Home n = 189	Total n = 383	
	n (%)	n (%)	n (%)	
Heart and vascular diseases	124 (63.9)	110 (58.2)	234 (61.1)	0.29
Rheumatic disease	124 (63.9)	121 (64)	245 (64)	>0.99
Endocrine diseases	36 (18.6)	35 (18.5)	71 (18.5)	>0.99
Lung diseases and allergies	29 (14.9)	20 (10.6)	49 (12.8)	0.22
Digestive system diseases	40 (20.6)	48 (25.4)	88 (23)	0.25
Kidney diseases	37 (19.1)	20 (10.6)	57 (14.9)	0.02
Skin diseases	24 (12.4)	18 (9.5)	42 (11)	0.42
Cancers	19 (9.8)	21 (11.1)	40 (10.4)	0.74
Other diseases	57 (29.4)	70 (37)	127 (33.2)	0.13
Depressive disorders	45 (23.2)	32 (16.9)	77 (20.1)	0.16
Anxiety disorders	73 (37.6)	41 (21.7)	114 (29.8)	0.001
Psychotic disorders	12 (6.2)	7 (3.7)	19 (5)	0.35
Insomnia	67 (34.5)	77 (40.7)	144 (37.6)	0.25
Psycho-organic syndrome (dementia)	14 (7.2)	24 (12.7)	38 (9.9)	0.09
Alcoholism	14 (7.2)	24 (12.7)	38 (9.9)	0.09

* Fisher’s exact test.

**Table 3:** Frequency of use of psychotropic drugs and anxiolytics, prescription of medications and frequency of medical examinations between respondents living at home and respondents living in retirement homes.

Prescribing and Use of Medications, Medical Examinations	Place of Residence			*p* Value *
	Home n = 194	Retirement Home n = 189	Total n = 383	
	n (%)	n (%)	n (%)	
**Use of psychotropic drugs**	110 (56.7)	108 (57.1)	218 (56.9)	>0.99
Prescription of medications				0.76
Primary care physician	161 (83)	159 (84.1)	320 (83.6)	
Specialist—psychiatrist	33 (17)	30 (15.9)	63 (16.4)	
Use of anxiolytics				0.05
Never	76 (39.2)	90 (47.6)	166 (43.3)	
Occasionally	57 (29.4)	60 (31.7)	117 (30.5)	
Permanently	61 (31.4)	39 (20.6)	100 (26.1)	
Length of use of anxiolytics				0.69
Less than 3 months	79 (40.7)	83 (43.9)	162 (42.3)	
3–6 months	5 (2.6)	5 (2.6)	10 (2.6)	
6–12 months	9 (4.6)	4 (2.1)	13 (3.4)	
1–5 years	10 (5.2)	12 (6.3)	22 (5.7)	
Longer than 5 years	38 (19.6)	39 (20.6)	77 (20.1)	
Medical examination				0.23
Never	45 (23.2)	47 (24.9)	92 (24)	
Occasionally	63 (32.5)	74 (39.2)	137 (35.8)	
Regularly	86 (44.3)	68 (36)	154 (40.2)	

* χ^2^ test.

**Table 4:** Peculiarities related to taking anxiolytics (CAGE scale).

CAGE Scale	Place of Residence			*p* Value *
	Home n = 194	Retirement Home n = 189	Total n = 383	
	n (%)	n (%)	n (%)	
Feeling the need to reduce use of tranquilizers or sleeping pills	149 (76.8)	164 (86.8)	313 (81.7)	0.01
They mind other people warnings about having issues with medication use	143 (73.7)	161 (85.2)	304 (79.4)	0.008
Feeling bad or guilty for use of tranquilizers	180 (92.8)	174 (92.1)	354 (92.4)	0.85
Sometimes the first thing they do after waking up in the morning is to drink tranquilizers	139 (71.6)	132 (69.8)	271 (70.8)	0.74

* Fisher’s exact test.

**Table 5:** Frequency of use of psychotropic drugs and anxiolytics, drug prescription, and frequency of medical examinations in relation to prevalence of clinical symptoms of addiction to anxiolytics.

Examined Variables	Prevalence of Clinical Symptoms of Addiction			*p* Value *
	No Symptoms n = 303	Significant Symptoms n = 80	Total n = 383	
	n (%)	n (%)	n (%)	
**Use of psychotropic drugs**	151 (49.8)	67 (83.8)	218 (56.9)	<0.001
Drug prescription				0.06
Primary care physician	259 (85.5)	61 (76.3)	320 (83.6)	
Specialist—psychiatrist	44 (14.5)	19 (23.8)	63 (16.4)	
Use of anxiolytics				<0.001
Never	153 (50.5)	13 (16.3)	166 (43.3)	
Occasionally	79 (26.1)	38 (47.5)	117 (30.5)	
Permanently	71 (23.4)	29 (36.3)	100 (26.1)	
Length of use of anxiolytics				<0.001
Less than 3 months	151 (49.8)	11 (13.8)	162 (42.3)	
3–6 months	7 (2.3)	3 (3.8)	10 (2.6)	
6–12 months	9 (3)	4 (5)	13 (3.4)	
1–5 years	15 (5)	7 (8.8)	22 (5.7)	
Longer than 5 years	51 (16.8)	26 (32.5)	77 (20.1)	
Medical examination				0.02
Never	82 (27.1)	10 (12.5)	92 (24)	
Occasionally	105 (34.7)	32 (40)	137 (35.8)	
Regularly	116 (38.3)	38 (47.5)	154 (40.2)	

* χ^2^ test.

**Table 6:** Perceived quality of life in relation to gender according to use of anxiolytics in respondents living at home or in retirement homes.

Areas of Life Quality (WHOQOL Scale)	Perceived Quality of Life		Total	*p* Value *
	Male	Female		
	Me (IQR)	Me (IQR)	Me (IQR)	
**Living at home**				
Physical health	57.1 (42.9–71.4)	57.1 (42.9–71.4)	57.1 (42.9–71.4)	0.88
Mental health	70.8 (50–75)	66.7 (50–75)	68.8 (50–75)	0.91
Social functioning	58.3 (50–66.7)	66.7 (50–75)	66.7 (50–75)	0.83
Environment	67.9 (57.1–75)	71.4 (62.5–75)	69.6 (60.7–75)	0.79
Total WHOQOL	64.3 (52.7–72.2)	66.5 (52.1–71.5)	65.1 (52.5–71.7)	0.93
**Living in retirement home**				
Physical health	51.8 (44.6–59.8)	62.5 (53.6–75)	60.7 (50–75)	0.02
Mental health	54.2 (38.5–62.5)	66.7 (54.2–75)	66.7 (50–75)	0.01
Social functioning	50 (35.4–56.3)	58.3 (50–75)	58.3 (50–75)	0.004
Environment	64.3 (51.8–75)	75 (63.4–82.1)	75 (60.7–82.1)	0.09
Total WHOQOL	57 (44.9–60.4)	66 (58.4–75.7)	64.5 (55.1–74.5)	0.003

Me (IQR) = median (interquartile range); * Mann–Whitney U test.

**Table 7:** Quality of life of respondents in relation to prevalence of clinical symptoms of addiction to anxiolytics.

Areas of Life Quality (WHOQOL Scale)	Perceived Quality of Life			*p* Value *
	No Symptoms (n = 303)	Significant Symptoms n = 80	Total n = 383	
	Me (IQR)	Me (IQR)	Me (IQR)	
Physical health	53.6 (39.3–67.9)	39.3 (28.6–43.8)	53.6 (38.4–67.9)	<0.001
Mental health	58.3 (41.7–70.8)	41.7 (33.3–50)	58.3 (41.7–70.8)	<0.001
Social functioning	58.3 (41.7–66.7)	45.8 (33.3–58.3)	58.3 (41.7–66.7)	0.003
Environment	67.9 (54.5–75)	62.5 (49.1–71.4)	67.9 (53.6–75)	0.04
Total WHOQOL	60 (47.8–69.7)	46.7 (40.7–54.2)	58.6 (46.7–68.5)	<0.001

Me (IQR) = median (interquartile range); * Mann–Whitney U test.

**Table 8:** Perceived quality of life of respondents living at home or retirement home in relation to prevalence of clinical symptoms of addiction to anxiolytics.

Areas of Life Quality (WHOQOL Scale)	Perceived Quality of Life			*p* Value *
	No Symptoms (n = 303)	Significant Symptoms n = 80	Total n = 383	
	Me (IQR)	Me (IQR)	Me (IQR)	
**Living at home**				
Physical health	53.6 (35.7–67.9)	39.3 (28.6–42.9)	53.6 (38.4–67.9)	0.007
Mental health	62.5 (45.8–70.8)	50 (33.3–54.2)	58.3 (41.7–70.8)	0.02
Social functioning	58.3 (41.7–66.7)	33.3 (33.3–50)	58.3 (41.7–66.7)	0.003
Environment	67.9 (53.6–75)	64.3 (57.1–71.4)	67.9 (53.6–75)	0.35
Total WHOQOL	59.4 (47.8–68.3)	46.6 (41.5–53.1)	58.6 (46.7–68.5)	0.006
**Living in retirement home**				
Physical health	57.1 (39.29–67.9)	39.3 (25–50)	53.6 (38.4–67.9)	0.04
Mental health	58.3 (41.7–70.8)	37.5 (29.2–45.8)	58.3 (41.7–70.8)	0.001
Social functioning	58.3 (41.7–66.7)	58.3 (33.3–66.7)	58.3 (41.7–66.7)	0.23
Environment	67.9 (57.1–78.6)	60.7 (46.4–71.4)	67.9 (53.6–75)	0.06
Total WHOQOL	60.7 (47.9–70.1)	46.7 (34.4–57.4)	58.6 (46.7–68.5)	0.02

Me (IQR) = median (interquartile range); * Mann–Whitney U test.

**Table 9:** Predictors of poorer quality of life (multivariate logistic regression).

Predictors	ß	*p* Value	OR (95% CI)
**All respondents**			
Significant symptoms (CAGE)	0.97	<0.001	2.63 (1.51–4.59)
Level of education—secondary	−0.53	0.03	0.59 (0.36–0.96)
Length of use of anxiolytics—3–6 months	2.47	0.003	2.30 (2.30–60.1)
No use of psychotropic drugs	−0.59	0.03	0.56 (0.33–0.93)
Medication prescription—specialist—psychiatrist	1.30	<0.001	3.66 (1.98–6.78)
Constant	−0.76	<0.001	
**Living at home**			
Significant symptoms (CAGE)	1.13	0.003	3.1 (1.46–6.56)
Level of education—secondary	−1.31	<0.001	0.27 (0.13–0.58)
Length of use of anxiolytics—3–6 months	2.86	0.02	17.5 (1.65–184.3)
Medication prescription—specialist—psychiatrist	1.58	<0.001	4.87 (2.02–11.75)
Constant	−0.80	<0.001	
**Living in retirement home**			
Significant symptoms (CAGE)	1.09	0.009	2.98 (1.32–6.77)
Length of use of anxiolytics—3–6 months	2.47	0.03	11.9 (1.21–16.8)
Length of use of anxiolytics—longer than 5 years	0.93	0.02	2.54 (1.19–5.41)
Medication prescription—specialist—psychiatrist	1.51	<0.001	4.54 (1.87–10.98)
Constant	−1.49	<0.001	

OR = odds ratio.

## Data Availability

All data generated analyzed during the current study are available from the corresponding author on reasonable request.
